# The expression of the receptor for advanced glycation end-products (RAGE) in RA-FLS is induced by IL-17 via Act-1

**DOI:** 10.1186/ar3398

**Published:** 2011-07-12

**Authors:** Yu-Jung Heo, Hye-Jwa Oh, Young Ok Jung, Mi-La Cho, Seon-Yeong Lee, Jun-Geol Yu, Mi-Kyung Park, Hae-Rim Kim, Sang-Heon Lee, Sung-Hwan Park, Ho-Youn Kim

**Affiliations:** 1 The Rheumatism Research Center, Catholic Research Institute of Medical Science, The Catholic University of Korea, Seoul, 505 Banpo-dong, Seocho-gu, Seoul 137-040, South Korea; 2 Division of Rheumatology, Department of Internal Medicine, Hallym University Kang-Nam Sacred Heart Hospital, Seoul, 143-729, Korea; 3 Division of Rheumatology, Department of Internal Medicine, Konkuk University School of Medicine, 4-12, Hwayang-dong, Gwangjin-gu, Seoul, 143-729, Korea; 4 Immune Tolerance Research Center, Convergent Research Consortium for Immunologic disease (CRCID), The Catholic University of Korea College of Medicine, St Mary's Hospital, 505 Banpo-dong Seocho-Gu, Seoul, 137-701, Korea

## Abstract

**Introduction:**

The receptor for advanced glycation end-products (RAGE) has been implicated in the pathogenesis of arthritis. We conducted this study to determine the effect of interleukin (IL)-17 on the expression and production of RAGE in fibroblast-like synoviocytes (FLS) from patients with rheumatoid arthritis (RA). The role of nuclear factor-κB (NF-κB) activator 1 (Act1) in IL-17-induced RAGE expression in RA-FLS was also evaluated.

**Methods:**

RAGE expression in synovial tissues was assessed by immunohistochemical staining. RAGE mRNA production was determined by real-time polymerase chain reaction. Act-1 short hairpin RNA (shRNA) was produced and treated to evaluate the role of Act-1 on RAGE production.

**Results:**

RAGE, IL-17, and Act-1 expression increased in RA synovium compared to osteoarthritis synovium. RAGE expression and production increased by IL-17 and IL-1β (*P <0.05 vs. untreated cells) treatment but not by tumor necrosis factor (TNF)-α in RA-FLS. The combined stimuli of both IL-17 and IL-1β significantly increased RAGE production compared to a single stimulus with IL-17 or IL-1β alone (*P <*0.05 vs. 10 ng/ml IL-17). Act-1 shRNA added to the RA-FLS culture supernatant completely suppressed the enhanced production of RAGE induced by IL-17.

**Conclusions:**

RAGE was overexpressed in RA synovial tissues, and RAGE production was stimulated by IL-17 and IL-1β. Act-1 contributed to the stimulatory effect of IL-17 on RAGE production, suggesting a possible inhibitory target for RA treatment.

## Introduction

Rheumatoid arthritis (RA) is a systemic autoimmune disease characterized by chronic synovial inflammation, which ultimately leads to the destruction of cartilage and bone in the affected joints. Synovial hyperplasia is a hallmark pathology of RA, and fibroblast-like synoviocytes (FLS) play a critical role in RA pathogenesis by producing pro-inflammatory soluble factors or activating other immune cells.

The receptor for advanced glycation end-products (RAGE) is a novel receptor that binds products of nonenzymatic glycation of proteins or advanced glycation end-products (AGEs) [1]. AGEs are a heterogeneous group of irreversible products formed from the nonenzymatic reaction of reducing sugars [2]. AGEs accumulate under a wide variety of biological conditions, such as diabetes, renal failure, aging, and inflammation [3]. The interaction of AGE and RAGE has been implicated in the activation of inflammatory signaling cascades and sequelae of AGE accumulation, such as diabetic complications, amplification of inflammation, and tissue injury [3]. AGEs cannot be removed until the protein degrades, and they alter tissue integrity and metabolism. Several receptors for the AGEs are known, and RAGE is a central signal transduction receptor for AGEs. RAGE is a member of the superfamily of immunoglobulin type cell surface receptors [4]. This receptor is strongly activated by cross-linked AGE-modified proteins. The activation of RAGE results in activation of an inflammatory signaling cascade, and up-regulation of RAGE is associated with sustained cellular perturbation and tissue injury [5]. Up-regulation of RAGE has also been reported under various pathologic conditions, such as vascular injury, diabetes, neurodegenerative disorders, and inflammatory diseases [6]. Overexpression of RAGE is implicated in the pathogenesis of RA. RAGE is overexpressed in synovial macrophages obtained from patients with RA, and synovial tissue cell culture supernatants strongly induce cell surface RAGE [7]. The increased level of RAGE pro-inflammatory ligands, such as high-mobility group box chromosomal protein 1 (HMBG-1) and S100/calgranulin in serum and synovial fluid in patients with RA may contribute to RAGE up-regulation [8,9].

Interleukin (IL)-17 and its major cell source, the type 17 T helper cells (Th17), have been implicated in the pathogenesis of various inflammatory diseases [10,11]. IL-17 mediates inflammatory responses including angiogenesis, recruitment of inflammatory cells, and induction of pro-inflammatory mediators in endothelial and epithelial tissues [12]. An up-regulated Th17 response or increased IL-17 production is associated with the pathogenesis of autoimmune diseases and chronic inflammation, including RA [13,14]. IL-17 mediates crucial cross talk between the immune system and tissues. Signaling through IL-17 receptors on synoviocytes induces immune cells to produce inflammatory factors such as IL-1 and IL-6 [15]. Many studies have been conducted regarding signaling molecules under IL-17 receptors, and nuclear factor-κB (NF-κB) activator 1 (Act1) is considered an essential protein for linking IL-17 receptors and downstream signaling pathways. Act1 is a recently identified 60-kD cytoplasmic adaptor protein that activates IκB kinase (IKK), liberating NF-κB from its complex with IκB [16].

We investigated whether pro-inflammatory cytokines, including IL-1, tumor necrosis factor (TNF)-α, and especially IL-17, can induce RAGE expression and production in RA-FLS. We also determined whether the stimulatory effect of IL-17 on RAGE is mediated by Act-1.

## Materials and methods

### Patients

Human FLSs were isolated from synovial tissues from patients with RA (F/M 7/1, median age 56 (range 26 to 65)), and patients with OA (F/M 6/1, median age 64 (range 46 to 71)) at the time of knee-joint arthroscopic synovectomy, as described previously [17]. The RA patients were all taking DMARDs (disease modifying anti-rheumatic drugs) and the rheumatoid factor was positive in five patients. ESR (erythrocyte segmentation rate), and CRP (C-reactive protein) checked pre-operatively were median 34 (range: 12 to 84) mm/hr and median 1.22 (range: 0.08 to 5.94) mg/dL respectively. The diagnosis of RA was confirmed by the revised criteria of the American College of Rheumatology [18]. Informed consent was provided according to the Declaration of Helsinki and obtained from all patients. Approval by the ethical committee of the Seoul St. Mary's Hospital (Seoul, Korea) was obtained.

### Isolation and culture of FLS

Synoviocytes were isolated by enzymatic digestion of synovial tissue specimens obtained from patients with RA undergoing total joint replacement surgery. The tissue samples were minced into 2- to 3-mm pieces and treated for four hours with 4 mg/ml type I collagenase (Worthington Biochemical Company, Freehold, NJ, USA) in Dulbecco's modified Eagle's medium (DMEM) at 37°C in 5% CO_2_. Dissociated cells were then centrifuged at 500 × *g *and resuspended in 10% fetal bovine serum in DMEM. After an overnight culture, the non-adherent cells were removed, and the adherent cells were cultured in DMEM supplemented with 20% fetal calf serum. Synoviocytes from passages 4 to 8 were used in each experiment. The RA-FLS were incubated with IL-17, IL-1β, or TNF-α (R&D Systems, Minneapolis, MN, USA) alone and in combination. To evaluate signal transduction, the RA-FLS were pretreated with 20 μM LY294002, 50 μM AG490, 10 μM SB203580, 20 μM PD98059, 10 μM parthenolide, or 10 μM curcumin and then treated with IL-17 for 12 h. The inhibitors were purchased from Calbiochem (Schwalbach, Germany).

### Immunohistochemistry of RA synovium and FLS

Immunohistochemical staining was performed on sections of synovium. Briefly, the synovial samples were obtained from eight patients with RA and one patient with osteoarthritis (OA) and fixed in 4% paraformaldehyde solution overnight at 4°C, dehydrated with alcohol, washed, embedded in paraffin, and sectioned into 7-μm-thick slices. The sections were depleted of endogenous peroxidase activity by adding methanolic hydrogen peroxide (H_2_O_2_) and were blocked with normal serum for 30 minutes. After an overnight incubation at 4°C with goat anti-human RAGE, anti-Act1 antibody (Santa Cruz Biotechnology, Santa Cruz, CA, USA) and antihuman IL-17 antibody (R&D Systems), pS727-STAT3, p-AKT, and p-C-Jun (Cell Signaling Technology, Danvers, MA, USA), the samples were incubated with the secondary antibodies, biotinylated anti-goat IgG and anti-rabbit IgG for 20 minutes. The sections were then incubated with streptavidin-peroxidase complex (Vector Laboratories Ltd., Peterborough, UK) for one hour followed by incubation with 3, 3-diaminobenzidine (DAKO, Glostrup, Denmark). The sections were counterstained with hematoxylin, and the samples were photographed with a photomicroscope (Olympus, Tokyo, Japan). Infiltrated inflammation cells of synovium histology grading system are classified and 400 magnification microscope observations set the number of positive cells at the site. We used the immunohistological criteria for classification of synovial tissues into "mild" and "severe". We evaluated the severity by the method presented in reference 20.

Dual immunohistochemical labelling (RAGE and CD55, CD68, P-STAT3, P-IKB, P-C-JUN, P-AKT) was performed using the DakoCytomation EnVision Doublestain-Kit (code K1395; DAKO North America, Inc. Carpinteria, CA, USA) according to the manufacturer's instructions [19]. In brief, the synovial tissue was incubated with the first primary antibody (anti-RAGE, Santa Cruz Biotechnology, Inc) and polymer method, developing the final color product using AEC (DAKO). The second primary antibody (anti-CD55, Serotec, Kidlington, Oxford, UK to detect fibroblast-like synoviocytes (FLS); anti-CD68, DAKO to detect macrophages; anti p- STAT3, p-IKB, p-c JUN, p-AKT) was placed on the sections at RT for one hour, followed by a standard immunohisto-chemical alkaline phosphatase method, to develop a color reaction with fast blue. No counterstain was used and the sections were mounted in an aqueous mounting medium. Samples were photographed with an Olympus photomicroscope (Tokyo, Japan)

### Real-time PCR for RAGE and Act-1

After the incubation, total mRNA was extracted from RA-FLS using RNAzol-B (Biotecx, Houston, TX, USA) according to the manufacturer's instructions. Reverse transcription of 2 μg of total mRNA was conducted at 42°C using the Superscript reverse transcription system (Takara, Shiga, Japan). Expression of the RAGE and Act-1 was determined by real time PCR with SYBR Green I (Roche Diagnostic, Mannheim, Germany). Each quantitative real-time PCR reaction was performed using 10 μL of SYBR green reaction mix (TAKARA SYBR Premix; Takara, Shiga, Japan), 200 nM of each primer RAGE and Act, 2 μL of template, and made up to 20 μL with sterile water in capillary tubes. All real-time reactions (standards, unknown samples, and controls) were performed in triplicate. The following primers were used for each molecule: for RAGE, 5'-CAG-TAG-CTC-CTG-GTG-GAA-CCG-TAA-C-3' (sense) and 5'-CCT ATC TCA GGG AGG ATC AGC ACA G-3' (antisense); for Act-1, 5'-GCA TTC CTG TGG AGG TTG AT-3' (sense) and 5'- GTC TCC GGA GGA ATT GTG AA-3' (antisense); for β-actin, 5'-GGA CTT CGA GCA AGA GAT GG-3' (sense) and 5'-TGT GTT GGC GAT CAG GTC TTT G-3' (antisense) in a LightCyclerÔ (Roche Diagnostics, Mannheim, Germany). The relative expression levels were calculated by normalizing the targets to the endogenously expressed housekeeping gene (b-actin). Melting curve analysis was performed immediately after the amplification protocol under the following conditions: 0 s (hold time) at 95°C, 15 s at 65°C, and 0 s (hold time) at 95°C. The temperature change rate was 20°C/s except in the final step, when it was 0.1°C/s. The crossing point (*C*_p_) was defined as the maximum of the second derivative from the fluorescence curve.

### Transfection of Act-1 short hairpin RNA (shRNA)

A hairpin oligonucleotide sequence targeting human ACT-1 (target sequence: 5'-GAGGCATTGATATCATTAA-3') was purchased from Dharmacon (Rockford, IL, USA). RA-FLS were plated in 60-mm dishes and transfected with 100 nM shRNA or 100 nM negative control vector using HiPerFect Transfection Reagent (Qiagen, Valencia, CA, USA), according to the manufacturer's protocol.

### Western blot for RAGE, signal transduction molecules, and their phosphor form

RA-FLS were incubated with LY294002, partherolide, or AG490 in the presence or absence of 10 ng/ml IL-17. After a one-hour culture, the cells were lysed. Protein concentrations in the supernatants were determined using the Bradford method (Bio-Rad, Hercules, CA, USA). Protein samples were separated with 10% sodium dodecyl sulfate-polyacrylamide gel electrophoresis and transferred to a nitrocellulose membrane (Amersham Pharmacia, Piscataway, NJ, USA). For Western hybridization, the membrane was pre-incubated with skim milk buffer (0.1% skim milk in 0.1% Tween 20 in Tris-buffered saline) for two hours, followed by incubation in primary Akt antibodies, phosphorylated Akt, IκB-α, phosphorylated IκB-α, STAT3, phosphorylated STAT3, c-Jun, phosphorylated c-Jun (Cell Signaling Technology), or RAGE (Santa Cruz Biotechnology) for one hour at room temperature. Horseradish peroxidase-conjugated secondary antibodies were added and the membranes were incubated for 30 minutes at room temperature. The hybridized bands were detected using the ECL detection kit and Hyperfilm-ECL reagents (Amersham Pharmacia).

### Determination of concentration of RAGE by sandwich enzyme-linked immunosorbent assays (ELISA)

The concentrations of RAGE in culture supernatants were measured using an enzyme-linked immunosorbent assay (ELISA) following the manufacturer's instructions (R&D Systems).

### Toxicity assessment of the stimulated RA-FLS

Toxicity of the stimulated RA-FLS was assessed using the lactate dehydrogenase (LDH) release assay. The cells were collected by centrifugation, and each pellet was mixed with 0.05% trypan blue. The proportion of cells containing trypan blue was determined microscopically. The LDH activity was measured in culture supernatants using the QuantiChromÔ lactate dehydrogenase kit (BioAssay Systems, Hayward, CA, USA) according to the manufacturer's protocol.

### Statistical analysis

All data are expressed as the mean ± SD. The statistical analysis was performed using SPSS 10.0 for Windows (SPSS Inc., Chicago, IL, USA). The differences between groups were analyzed using an unpaired Student's *t-*test, assuming equal variances. *P *< 0.05 was considered significant.

## Results

### Increased expression of RAGE, IL-17, and ACT- 1 in synovial tissues of patients with RA

**Figure 1:**
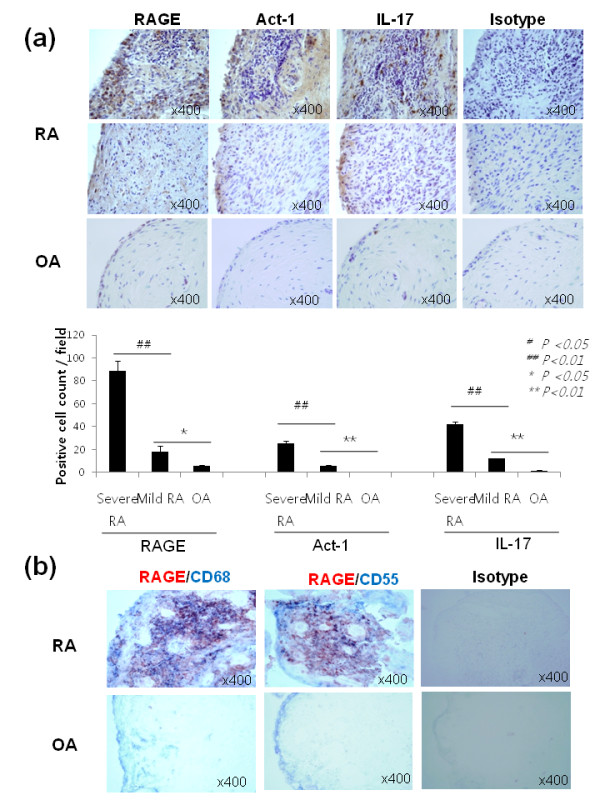
**Immunohistochemical staining of RAGE, Act1 and IL-17**. (**a**) The synovial tissue sections from patients with rheumatoid arthritis and osteoarthritis were stained with antibodies to RAGE, Act-1, IL-17, and H&E or an isotype-control antibody. The brown color shows the target. (**b**) Dual immunohistochemistry labeling using antibody RAGE and CD55 (for fibroblast like synoviocytes) or CD68 (for macrophages). All tissues were counterstained with hematoxylin (original magnification, x400).

The expression of RAGE, IL-17, and ACT-1 in synovial tissues from patients with RA (mild, severe) and patients with OA was examined by immunochemical staining. The immunohistochemical staining showed that RAGE, ACT-1, and IL-17 were expressed strongly in RA synovial tissues. In contrast, only scant expression of those molecules was observed in OA synovial tissues (Figure 1a). Strong RAGE expression was detected in the synovial lining and sublining layers and the perivascular area in RA synovial tissues. The severity of synovial inflammation was pathologically assessed [20]. Four synovial tissues showed mild degree inflammation and four showed severe inflammation. The positive cell count/field was evaluated. The positive cell count of RAGE, Act-1 and IL-17 was higher in synovial tissues with severe inflammation compared to synovial tissues with mild inflammation. The co-immunostaining of RAGE and surface markers of macrophage and FLS was performed. In RA synovial tissues, CD68 (macrophage marker) and CD55 (FLS marker) (blue) were co-stained with RAGE (red), which implies that RAGE was expressed by FLS and macrophages (Figure 1b).

### The stimulatory effects of IL-17 and IL-1β on RAGE production and expression in RA-FLS

**Figure 2:**
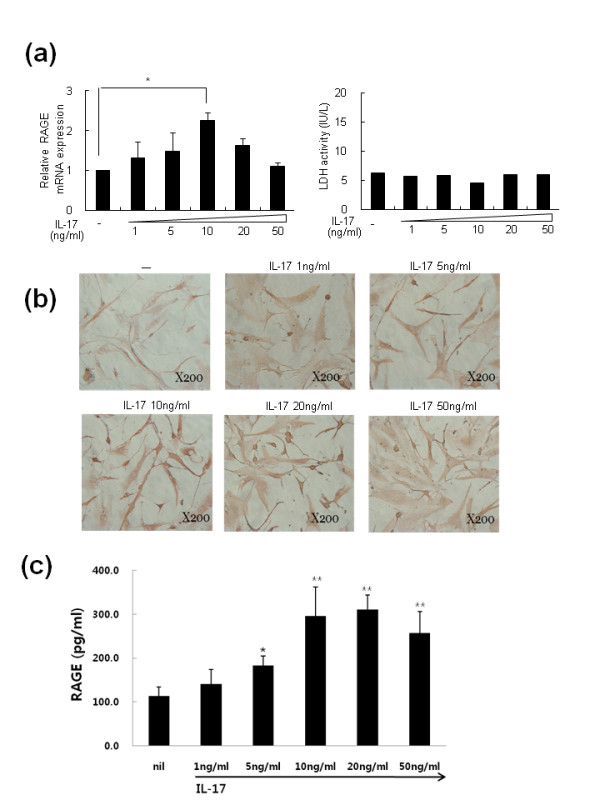
**The mRNA of RAGE was increased by IL-17 in a dose-dependent manner in rheumatoid arthritis fibroblast-like synoviocytes (RA-FLS)**. (**a**) RA-FLS were cultured with the indicated doses of IL-17 for 18 h. Total mRNA was extracted and analyzed by real-time PCR with SYBR Green I. Values are the mean ± SEM from one representative experiment with FLS from four patients with RA. RA-FLS (2 × 10^5^) were cultured with IL-17 for 18 h. Cell viability was assessed by lactate dehydrogenase (LDH) activity. (**b**) FLS were treated with the same method as (a). RAGE expression in the FLS was determined using a RAGE-specific antibody. (**c**) RA-FLS were cultured with the indicated doses of IL-17 for 48 h. RAGE was assessed by ELISA. Values are the mean ± SEM from one representative experiment with FLS from four patients with RA. *P < 0.05, **P < 0.01 compared to untreated cells.

Synovial fibroblasts obtained from patients with RA were incubated with various concentrations of IL-17. We observed that RAGE mRNA production measured by real-time PCR increased in RA-FLS following IL-17 treatment (Figure 2a). As shown in Figure 2a, RAGE expression was strongest when IL-17 was provided at 10 ng/ml (*P <0.05 vs. untreated cells) and gradually declined at higher doses. Cell cytotoxicity measured by LDH activity did not increase with IL-17 in culture supernatants. Increased RAGE expression was also observed with immunohistochemical staining or ELISA after 18 to 48 h of IL-17 treatment in the RA-FLS cultures (Figure 2b, 2c).

**Figure 3:**
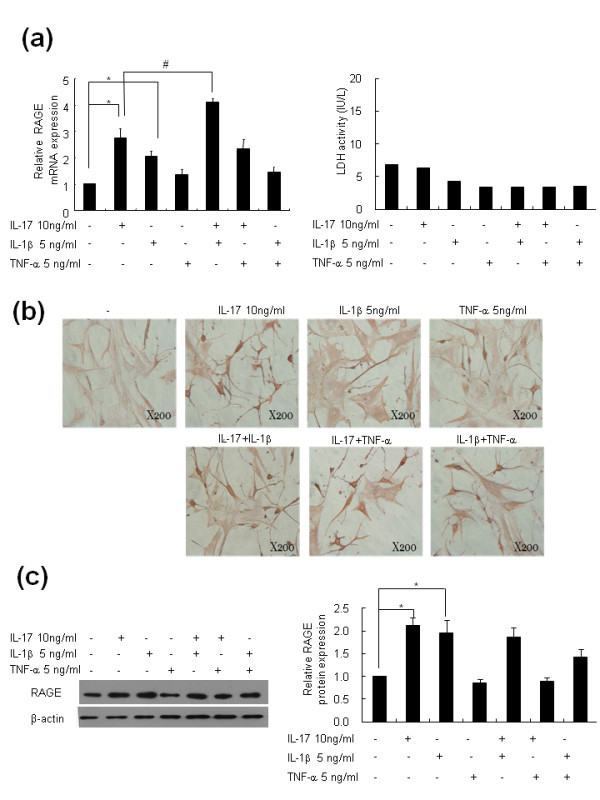
**IL-17 and IL-1β increased RAGE mRNA expression in RA-FLS**. (**a**) RA-FLS were cultured with 10 ng/ml IL-17, 5 ng/ml TNF-α, and 1 ng/ml IL-1β for 24 h, and RAGE mRNA was analyzed by real-time PCR. The lactate dehydrogenase (LDH) concentrations in the culture supernatants were determined by an activity assay kit. (**b**) RA-FLS were cultured as in Figure 3a. RAGE expression in the FLS was determined using a RAGE-specific antibody. The brown color shows the RAGE. (**c**) RAGE protein expression was identified by Western blot. Values are the mean ± SEM of triplicate cultures. *P < 0.05 compared to untreated cells and #*P *< 0.05 compared to IL-17-treated cells.

To evaluate the effects of other inflammatory cytokines and the combined stimuli of inflammatory cytokines on RAGE production in RA-FLS, FLS were cultured with IL-17 (10 ng/ml), TNF-α (5 ng/ml), and IL-1β (5 ng/ml) or a combination of those cytokines for 18 h (Figure 3a). RAGE mRNA expression was evaluated by real-time PCR. We observed that RAGE mRNA production increased with IL-17 and IL-1β treatment (*P <0.05 vs. untreated cells) but not by TNF-α. The combined stimuli of both IL-17 and IL-1β significantly increased RAGE production compared to IL-17 or IL-1β alone (#*P <*0.05 vs. IL-17 10 ng/ml). TNF-α did not show the additive effects on RAGE production induced by IL-17 or IL-1β. Immunohistochemical staining indicated that RAGE expression in RA-FLS also increased with IL-17, IL-1β, and the combined stimuli of IL-17 and IL-1β (Figure 3b). We also measured RA-FLS RAGE protein production by Western blot. IL-17 and IL-1β each enhanced RAGE protein production in RA-FLS. However, the combination of IL-17 and IL-1β did not show augmented effects on RAGE protein production (Figure 3c).

### IL-17-mediated RAGE induction in RA-FLS involves PI3 kinase, STAT3, NF-κB, and AP-1

**Figure 4:**
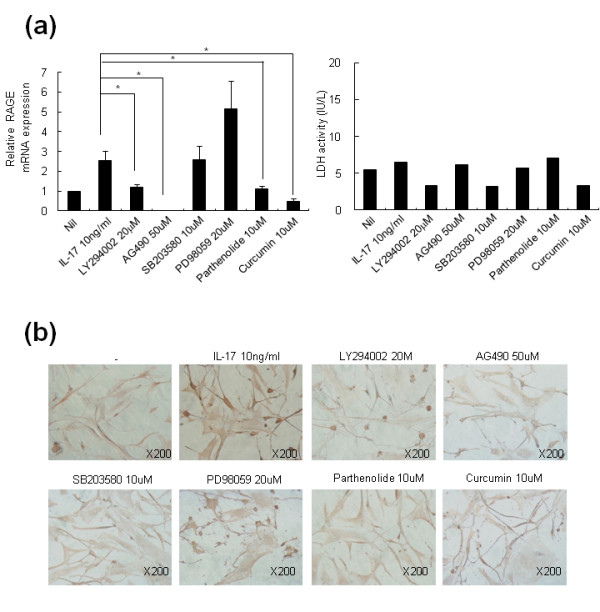
**IL-17-mediated RAGE induction in RA-FLS involves PI3 kinase, STAT3, NF-κB, and AP-1**. (**a**) RA-FLS were pretreated with 20 μM LY294002, 50 μM AG490, 10 μM SB203580, 20 μM PD98059, 10 μM parthenolide, or 10 μM curcumin for 30 minutes, and then 10 ng/ml IL-17 was added for 12 h. RAGE mRNA was analyzed by real-time PCR. RA-FLS were cultured as in Figure 4a. The lactate dehydrogenase (LDH) concentrations in the culture supernatants were determined using an activity assay kit. (**b**) FLS were treated with same method as (a). RAGE expression in the FLS was determined using a RAGE-specific antibody. The brown color shows the RAGE. Values are the mean ± SEM of triplicate cultures. *P < 0.05 compared to inhibitor-treated cells.

**Figure 5:**
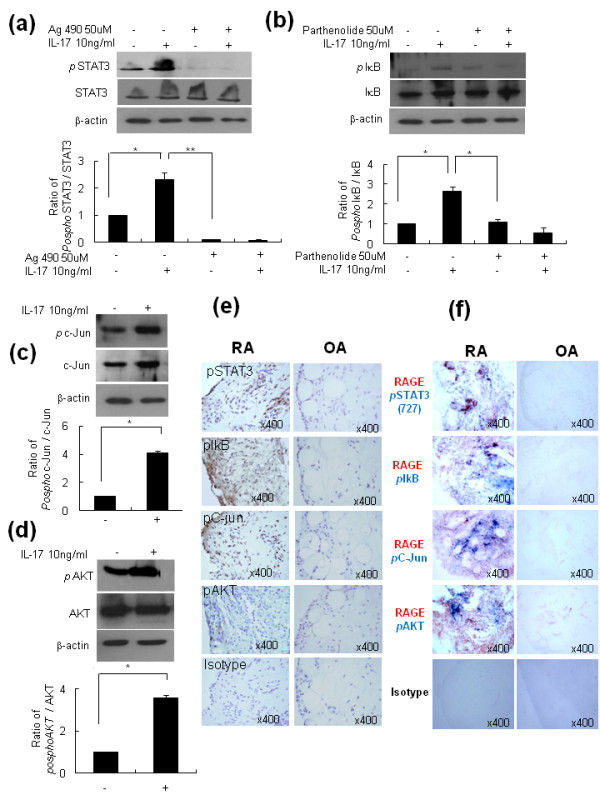
**IL-17-mediated-RAGE induction in RA-FLS involves PI3kinase, STAT3, NF-kB and AP-1**. RA-FLS were pretreated with 50 μM AG490 or 10 μM parthenolide for 30 minutes, and then 10 ng/ml IL-17 was added for 12 h. RA-FLS were cultured with 10 ng/ml IL-17. The protein levels of phosphoSTAT3, phosphoIkB, phosphoC-Jun, and phosphoAKT were analyzed by Western blot. The expression of phosphoSTAT3, phosphoIkB, phosphoC-Jun, and phosphoAKT on FLS was assessed by immunohistochemical staining using specific antibodies. Co-immunostaining of RAGE and phospho STAT3, phospho IκB, phospho c-Jun, and phospho AKT showed the link between *in vitro *signaling molecules and RAGE. Values are the mean ± SEM of triplicate cultures. *P < 0.05, **P < 0.005 compared to IL-17 or inhibitor-treated cells.

To evaluate the signal transduction pathways involved in the IL-17-mediated RAGE induction, RA-FLS were pretreated with 20 μM LY294002, 50 μM AG490, 10 μM SB203580, 1 μM PD98059, 10 μM parthenolide, or 10 μM curcumin, and the IL-17 induction of RAGE was evaluated. The inhibitory effects of various signal molecule inhibitors on the production of RAGE mRNA were assessed. LY294002, a phosphatidylinositol-3 kinase inhibitor, AG490, a STAT3 inhibitor, partherolide, an NF-κB inhibitor, and curcumin, an activator protein-1 (AP-1) inhibitor, showed inhibitory effects on the production of RAGE mRNA upon IL-17 stimulation (Figure 4a; *P <*0.05 vs. cells treated with IL-17 alone). In contrast, SB203580, a p38 MAPK inhibitor, and PD98059, a MEK1 inhibitor, failed to show inhibitory effects on IL-17-mediated RAGE mRNA induction. Immmunohistochemical staining showed the inhibitory effects of LY294002, AG490, partherolide, and curcumin on RAGE expression (Figure 4b). A Western blot and immunohistochemical staining of synovial tissues showed that IL-17 increased activation of phospho STAT3, phospho IκB, phospho c-Jun, and phospho AKT in RA-FLS (Figure 5). Co-immunostaining of RAGE and phospho STAT3, phospho IκB, phospho c-Jun, and phospho AKT showed the link between *in vitro *signaling molecules and RAGE (Figure 5f).

### Act-1 shRNA completely inhibited IL-17-induced RAGE production in RA-FLS

**Figure 6:**
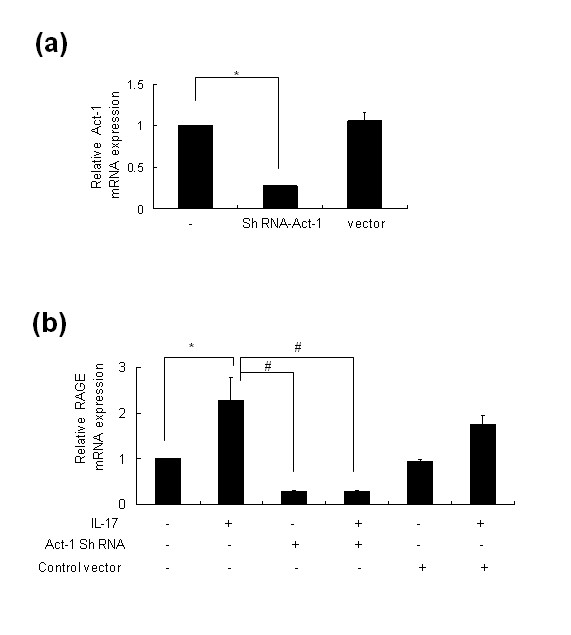
**Act1 shRNA completely inhibited IL-17-induced RAGE production in RA-FLS**. (**a**) RA-FLS were treated with Act-1 shRNA. Act-1 mRNA was analyzed by real-time PCR. (**b**) RA-FLS were pretreated with Act-1 shRNA for 24 h, and then 10 ng/ml IL-17 was added for 24 h. RAGE mRNA was analyzed by real-time PCR. *P < 0.05 compared to untreated cells and #*P *< 0.05 compared to IL-17 treated cells.

To identify whether Act-1 is involved in the signal pathway of IL-17-induced RAGE production and expression, we tested the effect of Act-1 shRNA on RAGE production. We produced Act-1 shRNA and confirmed the inhibitory effect of Act-1 shRNA on Act-1 expression (Figure 6a). Act-1 shRNA added to the RA-FLS culture supernatant completely suppressed the enhanced production of RAGE by IL-17 (Figure 6b).

## Discussion

An important role for RAGE has been reported in both OA and RA. In OA cartilage, an accumulation of AGE and up-regulation of RAGE were noted compared with normal healthy cartilage [21]. Inflammation-induced cartilage hypertrophy is induced by RAGE in OA [22]. In this study, we observed that RAGE expression was far stronger in RA synovium than in OA synovium. Drinda *et al*. also detected RAGE expression in the synovial lining, sublining, and stroma. In RA, many T cells (CD45RO(+)) and some macrophages (CD68(+)) showed positive immunostaining for RAGE, whereas B cells were mostly negative. They reported no difference in staining patterns between the RA and OA samples, which is not compatible with our observations [23]. The up-regulation of RAGE in RA synovium may be related to the abundance of inflammatory cytokines in RA synovial tissue. We observed that IL-1β and IL-17 have stimulatory effects on RAGE expression and production in RA-FLS. In contrast, TNF-α failed to show stimulatory effects on RAGE expression and production. The influence of inflammatory cytokines on RAGE expression in RA synovial tissue has been previously reported. Sunahori *et al*. reported that RAGE mRNA expression is augmented by various cytokines, most potently by IL-1β [7]. Notably, TNF-α, a central pro-inflammatory cytokine that plays important roles in RA pathogenesis, did not show strong effects on RAGE expression. In addition, the inducing effect of IL-17 on RAGE protein expression was inhibited by TNF-α (Figure 3c). This observation was compatible with a previous report by Sunahori *et al*. [7]. Although TNF-α may counteract the stimulatory effect of IL-17 on RAGE expression, in rheumatoid synovium, the expression of RAGE was increased as the final outcome as we observed in immunohistochemical staining of RA synovial tissues. IL-17 showed stimulatory effects on RAGE expression in FLS cultures in our experiments and may be relevant to the over-expression of RAGE on RA synovial tissues. However, the exact mechanism of RAGE over-expression in the milieu of various inflammatory cytokines of RA joints should be further investigated. This is the first report documenting the effect of IL-17 on RAGE expression in RA-FLS. The importance of IL-17 in RA pathogenesis has recently been emphasized. IL-17 stimulates the production and expression of pro-inflammatory cytokines from monocytes/macrophages [24] and from RA-FLS [25]. Furthermore, IL-17 contributes to angiogenesis [26] and osteoclastogenesis [27] in RA. Taken together, IL-17 contributes to RA pathogenesis due to perpetuations of inflammation to bone erosion and joint destruction. In our experiment, IL-17 induced RAGE production as well as RAGE mRNA expression in RA-FLS in a dose-dependent manner.

The engagement of RAGE stimulates diverse signaling cascades that regulate the adaptive and innate immune system [28]. Binding RAGE with its ligands activates NF-κB and results in subsequent activation of pro-inflammatory responses. Furthermore, the activation of NF-κB results in increased RAGE expression and increases the number of ligand binding sites, which in turn sustains NF-κB activation. The ability of RAGE to convert acute cellular activation into a sustained cellular response contributes to the development of complications in chronic diseases, such as diabetes and arthrosclerosis, and in neurodegenerative diseases [28]. In chronic inflammatory diseases such as RA, RAGE may contribute to the augmentation of the pro-inflammatory loop and sustain the inflammatory response. In our study, IL-17 was a strong inducer of RAGE in RA-FLS. IL-17 exerts an important role in inflammatory diseases both directly and indirectly. The up-regulation of RAGE is one of the functions of IL-17 for modulating the inflammatory condition.

We observed that Act-1 played an important role in IL-17-induced RAGE expression. Act-1 siRNA completely abrogated the IL-17-induced RAGE expression in our experiment. IL-17 activates the NF-κB and MAPK pathways and requires TNF receptor associated factor-6 to induce IL-6 [29]. The IL-17 receptor family shares sequence homology in their intracellular region with Toll-IL-1 receptor domains and with Act1. The Act1 and IL-17 receptors directly associate via a homotypic interaction and IL-17. Deficiency of Act1 in fibroblasts blocks IL-17-induced cytokine and chemokine expression. The absence of Act1 results in a selective deficiency of IL-17-induced activation of the NF-κB pathway [30]. We documented that the induction of RAGE by IL-17 was also Act-1 dependent in RA-FLS.

Blocking RAGE to attenuate diabetic complications and inflammation has been attempted. Soluble RAGE, a decoy receptor of RAGE, successfully blocks the binding of ligand and RAGE *in vitro *and *in vivo *[6,31]. Soluble RAGE reduces the complications of diabetes [31], suppresses Alzheimer pathology [32], and improves the outcome of experimental colitis [5]. Many studies have suggested that RAGE is a future target for treating chronic and inflammatory diseases. According to ELISA results, soluble RAGE level was also increased by IL-17 in our experiment. Soluble RAGE may act as a decoy receptor but we did not prove the function of soluble RAGE and cell surface RAGE. It should be further examined in future study. In our experiment, we determined that Act-1 could be a possible target regulating RAGE over-expression in RA. As IL-17 is important in the pathogenesis of various autoimmune diseases and chronic diseases, targeting Act-1 needs to be documented in other pathologic conditions.

## Conclusions

In this study, we found that RAGE up-regulation in RA-FLS was largely IL-17-dependent. As Act-1 is involved in IL-17-induced RAGE up-regulation, targeting Act-1 could be a promising target for regulating RAGE expression.

## Abbreviations

Act1: activator 1; AGEs: advanced glycation end-products; AP-1: activator protein-1; *C*_p_: crossing point; CRP: C-reactive protein; DMARDs: disease modifying anti-rheumatic drugs; ESR: erythrocyte segmentation rate; FLS: fibroblast-like synoviocytes; HMGB-1: high-mobility group box chromosomal protein 1; IKK: IκB kinase; IL-1β: interleukin-1beta; LDH: lactate dehydrogenase; MAPK: mitogen-activated protein kinase; NF-κB: nuclear factor-κB; OA: osteoarthritis; PBMC: peripheral blood mononuclear cell; PI3K: phosphatidylinositol (PI)-3 kinase; RA: rheumatoid arthritis; RAGE: receptor for advanced glycation end products; shRNA: short hairpin RNA; STAT3: signal transducer and activator of transcription 3; TNF: tumor necrosis factor.

## Competing interests

The authors declare that they have no competing interests.

## Authors' contributions

YJH, YOJ, and MLC contributed to conception and design, acquisition of data, analysis and interpretation of data, drafting of the article and final approval of the submitted manuscript. OHJ, JGY and SYL contributed to immunohistochemistry. HRK, MKP and SHL helped with PCR, Western blotting and ELISA, and SHP and HYK contributed cell culture and transfection. All authors approved the final manuscript.
